# Teleorthopedic: A Promising Option During and After the Coronavirus Disease 2019 (COVID-19) Pandemic

**DOI:** 10.3389/fsurg.2020.00062

**Published:** 2020-08-28

**Authors:** Michael Anthonius Lim, Raymond Pranata

**Affiliations:** Faculty of Medicine, Universitas Pelita Harapan, Tangerang, Indonesia

**Keywords:** telemedicine, telehealth, teleorthopedic, telerehabilitation, virtual consultation, virtual rehabilitation, coronavirus, COVID-19

The impact caused by the Coronavirus Disease 2019 (COVID-19) pandemic has been strong enough to force all aspects of medical services to innovate. Although initially call into doubt, virtual medical services emerge to be one of the most promising options for orthopedic services globally. Considering the widespread implementation of isolation, quarantine, and lockdown, the incidence of motor vehicle accidents and traumatic injuries is markedly reduced which leads to a decrease in emergency surgeries (e.g., limb-threatening conditions, bone or joint infections, compartment syndrome). Also, most elective procedures, including orthopedic treatments (e.g., total joint arthroplasty, chronic peripheral nerve compression syndrome, chronic ligament, and tendon injuries), are deferred indefinitely, resulting in a drastic reduction in orthopedic service (1). Since the number of new COVID-19 cases is still increasing worldwide, surgical activity poses another challenge for healthcare workers because it presents a great risk of nosocomial transmission. The gradual resumption of orthopedic surgery must be carefully planned to take into account the risks and benefits of each case (2).

Orthopedic surgery has the potential risk of severe acute respiratory syndrome coronavirus 2 (SARS-CoV-2) transmission from patient to surgeon and the team through inhalation of the infected virus in aerosols or surgical smokes (3). A careful selection of patients who are eligible for surgery should be implemented to minimize the risk of infections and complications. Preselection of prioritized patients should take into account several parameters including COVID-19 exposure status, age, comorbidities, American Society of Anesthesiologists (ASA) physical status classification system, and socio-professional condition (1). Regardless of the presence of symptoms, all candidates for surgery should be considered as possible COVID-19 cases and the use of personal protective equipment (PPE) is recommended for the surgical team. Given that the shortage of surgical team members is a major concern during such unusual circumstances, healthcare protocols related to disease prevention, mitigation, and containment must be strengthened, while screening and monitoring of COVID-19 for all operated patients are compulsory to limit the risk of contagion. The length of hospitalization should be minimized and healthcare visits must be minimized and turned into virtual meetings (2).

Economic crisis is experienced by many healthcare facilities, especially those who rely heavily on elective surgical procedures. After the COVID-19 pandemic, the return to normalcy may be different and slower than what we normally do, and the introduction of telemedicine and other internet-based medical services is suggested for the survival of healthcare system (4). Although many believe that orthopedic surgeons cannot assess their patients without a direct examination, the transformation from standard in-person appointments into virtual remote sessions is inevitable. Currently, audiovisual-assisted meetings have been incorporated into orthopedic practice given the evidence that they are as effective or at least not inferior to those obtained with conventional face-to-face visits (5). This new form of medical services facilitates the exchange of information between different parties, including specialists, general practitioners, physiotherapists, and patients (6). Virtual consultations and rehabilitation in orthopedic services have been proven to be cost-effective from the perspective of the community and medical providers and reported to increase patient satisfaction, reduce travel and save time for the patients, and minimize costs. Teleorthopedic practices were found to improve accessibility and quality of care, with the average quality-adjusted life years (QALYs) gained between the telemedicine group (0.09) and standard consultation group (0.05) observed to be not significantly different (*P* = 0.29) (7).

Patient-reported satisfaction and health outcomes were found to be not significantly different between audiovisual-guided sessions and traditional doctor-patient appointments, and a high proportion of patients preferred virtual meeting as their next consultation (8). Considering the satisfaction of patients and medical providers, as well as the economic benefits, teleorthopedic and telerehabilitation practices are certainly promising given the increasingly sophisticated medicine and technology. In the future, people might be able to track their vital signs or other biomarkers through the use of digital health programs and wearable sensor technologies that are connected to applications on the device (2). The delivery of orthopedic services at a distance is safe and effective with minimum pitfalls and undesirable events which will ultimately improve the effectiveness of rehabilitation after orthopedic surgery (5–7). This new type of orthopedic delivery bridges the problem of distance between patients, doctors, and physiotherapists, and eventually minimizes the risk of SARS-CoV-2 transmission by avoiding human contact. For patients who reside in remote areas, this online health services will facilitate the continuity of their medical care. The majority of healthcare workers agreed and championed the idea that audiovisual-guided sessions could aid them in providing continued care during the COVID-19 pandemic (4).

The classification of surgical procedures based on the degree of emergencies is highly important, with certain conditions (e.g., life-threatening conditions, major trauma cases) requiring direct examination and immediate action by the surgeons and nurses. However, some elective cases can be adequately managed through consultation and rehabilitation at a distance (1). During a virtual session, the doctor can explore the patient's medical history and ask them to perform some movements to complete the physical examination. Using a browser in any devices or computers, a ruler or goniometer applications can measure lengths or angles that assist the doctor evaluates the patient's range of motion. Requesting supporting investigations such as X-ray, ultrasonography (USG), magnetic resonance imaging (MRI), computed tomography (CT)-scan, or blood tests are also possible and some can even be done virtually. Teleradiology such as tele-USG has been very useful especially in diagnosing soft-tissue injuries. Medical practitioners from various specialties can communicate clearly via audiovisual platforms, and they can talk with the physiotherapists who manage their respective patients. Telemedicine will undoubtedly help medical providers in establishing a diagnosis, providing education, planning treatment, developing prevention, and completing a comprehensive follow-up or evaluation of patients' clinical outcomes (6).

In Indonesia, most orthopedic services are still provided through conventional face-to-face meetings, with many elective procedures being postponed indefinitely during such pandemic. A lot of people are still reluctant or afraid to visit hospital to seek treatment, while a portion of the population in the remote areas cannot reach healthcare facilities or depend on more traditional treatments, such as bone setters, soft tissue massage and manipulation, and traditional medicines (9). This is obviously worrying, because of the consequences of delayed management of trauma, fracture, or other musculoskeletal injuries (10). Virtual medical services are already available in many specialties, including orthopedic and rehabilitation, through applications on smartphones or computers. It is easy to use at a reasonable price range. However, many individuals still depend on hands-on assessment and treatment. At present, despite limitations due to COVID-19, teleorthopedic and telerehabilitation are not widely used by the general population.

During the COVID-19 lockdown, many individuals became accustomed to sedentary behavior since they spent more time at home or even worked from home. They rarely exercise or participate in sports activities partly because of the inability to go outside. Physical inactivity is a serious concern other than an ever-increasing pandemic, where a surge of non-communicable diseases can emerge shortly (11). Considering this possibility, people with comorbidities (e.g., diabetes, hypertension, lung disease, cardiac disease, cerebrovascular disease, renal, and liver problems), who are at high risk of developing severe COVID-19 with complications, will reap the benefits of a vast delivery of virtual care (12–21). The COVID-19 pandemic has accelerated telemedicine implementation in orthopedic and rehabilitation even though it was originally regarded as an impossibility. Online orthopedic and rehabilitation services will help the most chronically disabled individuals to exercise at home effectively and consistently (22).

Both types of telemedicine delivery, real-time (synchronous) and store-and-forward (asynchronous), bring significant benefits to patients undergoing rehabilitation (23). This technology-based medical service has emerged as a promising alternative to conventional face-to-face visits, especially for post-operative patients with limited mobility (24). Patients who undergo orthopedic surgery are advised to maintain a physically active lifestyle as it can enhance immunity, reduce the risk of contracting diseases, and eventually lower the risk of death (22). Individualized and interactive training session or self-directed physical therapy will likely be an attractive new rehabilitation method that is expected to promote sustainability (2). In the United States, Virtual Exercise Rehabilitation Assistant (VERA) has succeeded in creating animated trainers that provide physical therapy and exercise programs developed by doctors and physiotherapists specifically for each patient to train at home. Biometric data is utilized in designing programs so that appropriate, suitable, yet convenient rehabilitation is provided. This system allows healthcare providers to communicate clearly with their patients while continuously monitoring the progress and at the same time giving advice, correction, or feedback on their posture, movements, or performance. In terms of clinical outcomes, the virtual personal trainer was as effective and safe as the traditional counterpart (25, 26).

As with any new system, the practice of virtual examination and assessment in orthopedic and rehabilitation is not without problems. Technological constraints, as well as individual skepticism and reluctance, both from providers and patients, are considered as the major obstacles in delivering teleorthopedic and telerehabilitation (23). Many individuals still prefer standard in-person appointments and think that consultation, examination, and assessment cannot be performed well enough remotely. Cooperation with primary healthcare facilities at remote or rural areas can be useful because they can assist the communication process between specialized services (e.g., orthopedic, rehabilitation) and the patients (27). This is particularly important in developing countries, including Indonesia, where rural communities are unwilling to use advanced technology or even hesitate to seek appropriate medical service. In a 5.5 years review of the use of email-based teleorthopedic service in remote communities with limited access to care, this online practice allowed 79% of acutely injured patients to be treated locally, which significantly reduced travel time and expenses (28). Another limitation is the specific regulations set by health insurance providers, both public or private, that financially limits patient access to telemedicine (29).

COVID-19 is changing the delivery of orthopedic and rehabilitation care. In the future, there will be more virtual care. Designing standardized virtual examinations and measurements is the next move to enable doctors and physiotherapists to provide the same quality of medical services. Guidelines and recommendations for reinstalling orthopedic activity had been developed, including classification of procedures based on the emergency and urgency levels, stratification of the patient's risk of infection for the eligibility of surgery, recommendations for PPE use in the operating area, and strategy for resuming elective orthopedic surgery in four phases (1). Teleconsultation and telemonitoring are currently two of the most commonly used virtual orthopedic services (6). At present, machines operated by surgeons or robotic surgeons have been introduced and telesurgery is likely to be more popular and advanced in the future considering continuous medical and technological innovation. Telecommunication between surgeons and robots has become more sophisticated recently. However, we must ensure that the hacking of robotic surgery is not possible because this threat cannot be tolerated. An additional concern about the implementation of online medical services is that of privacy and data (23). Therefore, advancements in cyber-security and privacy must precede the development of medical technology and medical records and databases must be protected at all costs.

## Author Contributions

ML and RP confirm being the only contributors of this work and have approved it for publication. All authors contributed to the article and approved the submitted version.

## Conflict of Interest

The authors declare that the research was conducted in the absence of any commercial or financial relationships that could be construed as a potential conflict of interest.
